# Outcomes after Early Anticonvulsant Discontinuation in Aneurysmal Subarachnoid Hemorrhage

**DOI:** 10.4172/2329-6925.1000173

**Published:** 2015-01-24

**Authors:** Sherry Hsiang-Yi Chou, Julius Gene Silva Latorre, Gulhan Alpargu, Christopher S Ogilvy, Farzaneh A Sorond, Guy Rordorf

**Affiliations:** 1 Departments of Critical Care Medicine, Neurology, and Neurosurgery, University of Pittsburgh School of Medicine, USA; 2 Department of Neurology, SUNY Upstate Medical University, USA; 3 Department of Statistics, California State University Fullerton, USA; 4 Department of Neurosurgery, Massachusetts General Hospital, Harvard Medical School, USA; 5 Department of Neurology, Brigham and Women’s Hospital, Harvard Medical School, USA; 6 Department of Neurology, Massachusetts General Hospital, Harvard Medical School, USA

**Keywords:** Anticonvulsant, Subarachnoid hemorrhage, Vasospasm, Mortality

## Abstract

**Background:**

Empiric use of anticonvulsant (AED) for seizure prophylaxis in aneurysmal subarachnoid hemorrhage (SAH) remains controversial and may be associated with worse SAH outcome. We determined the safety and feasibility of early discontinuation of empiric AED in a select cohort of SAH patients.

**Methods:**

In a cohort of 166 consecutive SAH patients, a subset underwent early AED discontinuation if they were awake and following commands after aneurysm treatment. We examined the effect of AED discontinuation on seizure incidence, mortality and functional outcome at discharge using logistic regression and validated results using 70%-30% data partition.

**Results:**

Seventy-three subjects underwent AED discontinuation. Patient groups had similar gender, age, Fisher grade, incidence of craniotomy, vasospasm, ischemic infarct, intraventricular and intraparenchymal hemorrhages. Hunt-Hess (HH) grade were lower in AED-discontinuation group. Clinical or electrographic seizure occurred in 1/93 (1%) patients on AED and 0/73 patient in AED-discontinuation group. Crude mortality was 24% in patients on AED and 2.7% off AED. After adjusting for age, HH grade, vasospasm, ischemic infarct, intracerebral, and intraventricular hemorrhage, AED discontinuation remains independently associated with lower mortality and higher odds of discharge to home (p=0.0002). AED use is not associated with angiographic vasospasm on exploratory analysis.

**Conclusion:**

AED discontinuation in SAH patients who are awake and following commands post aneurysm treatment is safe, feasible, and associated with better outcome at hospital discharge. A larger, prospective study is necessary to determine if empiric AED use in SAH leads to poorer functional status.

## Introduction

Subarachnoid hemorrhage (SAH) affects 50,000 people yearly in the United States and affects up to 30/100,000 in other parts of the world such as Japan and Finland [1,2]. Though only 5% of all strokes, SAH is a highly morbid disease responsible for 27% of all stroke-related years of potential life lost before age 65 and leaves one third of survivors requiring lifelong care [3,4]. Reported incidence of SAH-related seizures varies widely but may be as high as 25% [5], prompting most clinicians to empirically treat all SAH patients with prophylactic anticonvulsant (AED). Despite newer studies reporting a much lower seizure incidences in SAH [6], approximately 65% of all SAH patients still receive empiric AED therapy [7]. To date, empiric AED use for seizure prophylaxis following SAH remains highly controversial and AED use in SAH is largely driven by physician preference [7–9]. Emerging data suggest empiric use of prophylactic AED post SAH, even for a short time, is associated with worse functional outcome [7,10,11]. On the contrary, certain AEDs have demonstrated neuroprotective properties in animal models in ischemic/hypoxic brain injury and in SAH [12,13], which argue for its potential beneficial effect in SAH in addition to seizure prevention. There has been no prospective clinical trial that examined the effect of prophylactic AED use and SAH outcome.

Prophylactic AED use also may not necessarily be effective in preventing new onset seizures and epilepsy. Prospective human studies have demonstrated that prophylactic AED use did not decrease the incidence of new onset seizures in patients with brain tumors [14] and in patients beyond 7 days after traumatic brain injury [15]. Whether prophylactic AED use is effective in preventing post-SAH seizures remains unknown. Meanwhile, AED use in critical illness and SAH is associated with significant morbidities, including drug hypersensitivity, hepatic impairment, drug-induced fever, drug-induced hyperammonemia [16], excessive somnolence, potentially reduction in therapeutic effect of nimodipine through up-regulation of hepatic P450 system [17], and functional and cognitive disability following SAH [10]. Reducing the duration of prophylactic AED such as phenytoin reduces drug-related complications [18]. In this study, we aim to determine whether early discontinuation of prophylactic AED use in a select group of critically-ill SAH patients is safe. Our primary outcomes of interest are the incidence of seizure following AED discontinuation and functional status at hospital discharge. We then explored in-hospital mortality and incidence of angiographic vasospasm as secondary safety outcomes.

## Materials and Methods

### Patient selection

We studied the effect of AED withdrawal in a cohort of consecutive SAH patients admitted to the Neuroscience Intensive Care Unit (NICU) at a single center over a 2 year period. We utilized the introduction of a new clinical protocol that selectively discontinued AED in SAH patients who met the following eligibility criteria: i) older than 18 years of age, ii) had aneurysmal SAH or pre-pontine SAH, iii) the bleeding aneurysm(s) (if present) had been secured via surgical or endovascular intervention, iv) patient had had more than 24 hours to recover from surgical or endovascular procedure for cerebral aneurysm treatment, and v) patient was awake and following commands. Patients were excluded from this protocol if i) they had secondary SAH due to trauma or vasculitis, ii) they had seizure-like events associated with onset or during early course of SAH, or iii) they had a known seizure disorder prior to SAH. For safety reasons, this protocol excluded patients with sustained ICP elevation (>20 cm H_2_O sustained for over 5 minutes) and patients with evidence of mass effect on brain computed tomography (CT) imaging. All patients, whether or not on prophylactic AED, were followed for development of clinical seizures during their hospitalization. Patients were considered to have a seizure if they manifested clinical events diagnosed as seizures by their treating physicians or if electrographic seizures were detected on electroencephalograms (EEG) performed for clinical indications. Continuous EEG monitoring was not performed for routine monitoring on SAH patients. The local institutional review board approved this study.

### Standardized clinical SAH treatment protocol

All SAH patients were treated according to a standardized clinical protocol which had been published previously [19]. All SAH patients underwent diagnostic cerebral angiography and all patients with intracerebral aneurysm present on angiography were treated with surgical clipping or endovascular coiling within 24 hours of presentation. SAH patients were monitored in the NICU for 10-14 days post hemorrhage for diagnosis and treatment of cerebral vasospasm, defined as changes in the neurological function attributable to cerebral ischemia (clinical vasospasm), peak systolic middle cerebral artery (MCA) velocity >200 cm/sec with a Lindegaard ratio >3 (TCD vasospasm), or evidence of focal or generalized reduction of cerebral arterial caliber on diagnostic cerebral CT angiography (CTA) or conventional cerebral angiography (angiographic vasospasm). All patients with clinical or TCD vasospasm underwent CTA or conventional cerebral angiography. Those with confirmed angiographic vasospasm were treated with intra-arterial calcium channel blocker injection.

### Anticonvulsant treatment and EEG evaluation protocol

All patients with aneurysmal SAH were loaded with intravenous phenytoin upon presentation to the hospital unless there is a known phenytoin allergy, in which case they were loaded with an alternative intravenous anticonvulsant. Patients who were already taking an AED prior to SAH are loaded with phenytoin or another intravenous AED while their home AED were continued and maintained at therapeutic levels. Serum phenytoin levels were monitored daily and phenytoin dose was modified to target a corrected serum phenytoin level of 10-20 mg/dL. Phenytoin was discontinued and switched levetiracetam if patient developed fever of unknown origin, skin rash, or hepatic dysfunction while receiving phenytoin. Serum levetiracetam levels were not monitored. All patients suspected to have clinical seizure activity were evaluated with video-EEG. Patients without overt clinical seizures but who had unexplained obtundation were also evaluated with EEG to rule out occult seizures and non-convulsive status epilepticus (NCSE). Patients with poor functional status were all evaluated with at least one routine EEG prior to prognostication and changes in goals of care.

### Data collection and definition of terms

We identified consecutive aneurysmal SAH patients admitted over a 2-year peroid through a prospective ICU database. We collected basic patient demographic data, clinical reports of seizures, EEGs results, AED use versus AED discontinuation, AED agent used, and prognostic data including Hunt and Hess grade, Fisher grade, presence of intraventricular hemorrhage (IVH) and intraparenchymal hemorrhage (IPH) on initial head CT upon hospital presentation, development of angiographic vasospasm, and presence of SAH-related ischemic infarct (acute or sub-acute hypodensity on head CT that developed at anytime during subject’s hospitalization for SAH). A single investigator reviewed all CT imaging for presence of ischemic infarct, ICH, and IVH. We recorded discharge status (home, other medical facility, or death). Patients with good neurological function at time of hospital discharge returned home. Patients with functional disabilities that prevent safe discharge to home were discharged to other medical facilities such as inpatient rehabilitation center or nursing home. Insurance status did not affect a patient’s eligibility for rehabilitation or nursing home care in this patient population.

### Statistical analysis

Categorical variables were compared using Chi-square or Fisher’s exact test as appropriate. Continuous variables with normal distribution were compared using a 2-tailed t-test. Continuous variables with nonnormal distribution were compared using the Mann-Whitney U test. Statistical analysis was performed using SAS 9.1 and Insightful Miner 7.0.

We evaluated the association between discharge status and AED discontinuation using logistic regression modeling. In order to evaluate the robustness of our results, we elected to partition the data set into a 70%-30% split using a random number generator. We built a logistic regression model for discharge functional status using the larger data subset (Training data set). Co-variates used to build this regression model were selected based on their univariate correlation with AED discontinuation and clinical outcome as well as knowledge of their clinical significance in SAH outcome. We then validated this logistic regression model using the smaller data subset (Testing data set) which was not used for model building. Somer’s D statistics for performance of regression model on training and on testing data sets were calculated using SAS. Graphical illustration of ROC for this logistic regression model on training and testing data set was generated using Insightful Miner.

We explored the effect of AED discontinuation on in-hospital SAH mortality and incidence of angiographic vasospasm using logistic regression modeling. These exploratory analyses were performed without data set partitioning. Co-variates used in these logistic regression models were selected based on univariate association as well as known clinical relevance. Automated variable selection algorithms were not used for any analysis. Age was included in all models as a continuous variable, and HH and Fisher grades were included as ordinal variables.

## Results

We identified 181 consecutive SAH patients over a 2-year period. Fifteen patients were excluded due to known seizure disorders or seizure-like event at onset of SAH. The remaining 166 patients were analyzed, of whom 9 did not receive AEDs during the entire hospitalization. These patients were included in the data analysis in the “off AED” group.

Ninety three patients remained on AED (on AED group) while 73 patients underwent early AED discontinuation (off AED group). Gender, mean age, Fisher grades, and incidence of craniotomy are comparable between these two groups (Table 1). In the off AED group, AED withdrawal occurred early in the hospitalization at ~ 48 hours post hospital presentation. Those patients were subsequently observed for an average length of stay of 15-16 days for incidence of seizure. A higher proportion of subjects who remained on AED had HH grade of 3 or above. Presence of intraventricular hemorrhage (IVH) and intraparenchymal hemorrhage (IPH), aneurysm location, and incidence of angiographic vasospasm and ischemic infarcts on CT were comparable between the two groups.

Ninety-eight percent of all patients who remained on AED were exposed to phenytoin during their NICU course (Table 2). Fifty-nine percent of subjects who remained on AED were switched from phenytoin to another AED.

### Seizure incidence

No one developed clinical or electrographic seizures post AED discontinuation. One patient who remained on AED experienced one generalized tonic clonic seizure (Table 3). No patient who underwent early AED withdrawal were put back AED prior to hospital discharge.

### Functional outcome at discharge

**Figure 1:**
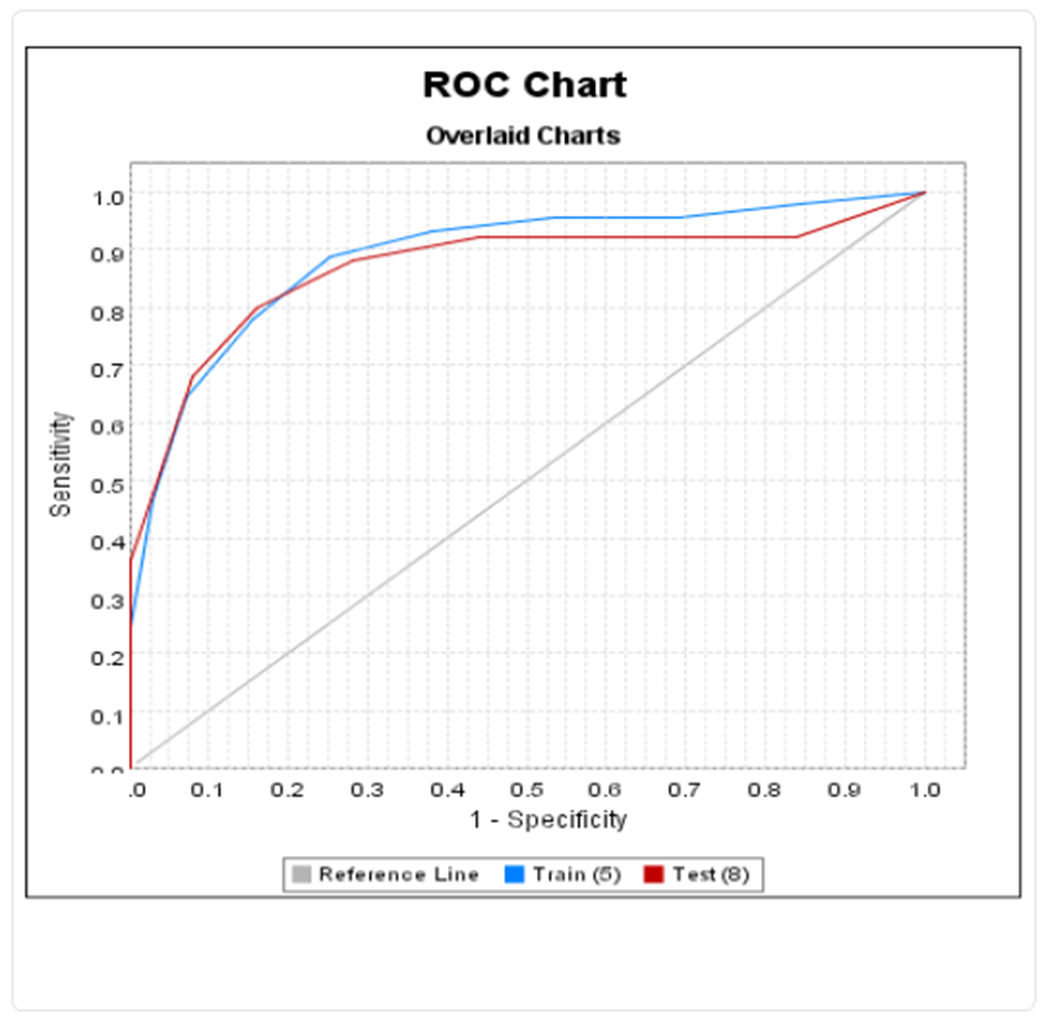
Receiver Operating Curves for Logistic Regression Model of Discharge Outcome Applied to Training and Testing Data Sets.

Early AED discontinuation was independently associated with higher odds of discharge to home after controlling for HH score, age, vasospasm, ischemic infarct, presence of IPH and IVH (Table 4). Higher HH score, older age, and presence of vasospasm and infarct on CT are associated with lower odds for discharge to home. This logistic regression model was validated using data partition with Somer’s D statistic of 0.894 on training data set and 0.864 on testing data set. Figure 1 illustrates the comparable receiver-operating curves (ROC) for this model using the training and testing data set.

### Mortality

Crude mortality was 24% in the on AED group versus 2.7% in the off AED group (Table 3). The two patients who died in the “off AED” group both presented with HH grade 5 SAH and died within 24 hours of hospital arrival after withdrawal of life-prolonging care. One of the two patients was never started on an AED. Based on pre-specified analysis criteria and to minimize selection bias, both of these subjects were analyzed as part of the “off AED” group though they did not meet clinical criteria to undergo early AED withdrawal. Of the 22 subjects who died in the on AED group, average survival was 7 days post SAH (range: 0-18 days) and 50% of all deaths occurred on or before day 6 post SAH. Nine of these deaths occurred after withdrawal of life-prolonging care. No death occurred due to acute cardiovascular collapse. AED discontinuation was independently associated with survival to hospital discharge after adjusting for age, HH grade, vasospasm, ischemic infarct, IPH and IVH (Table 4).

### Vasospasm

The crude rate of angiographic vasospasm was 30% in the ofFAED group versus 39% in the onAED group. Early AED discontinuation was not associated with angiographic vasospasm after adjusting for effects of age, HH grade, Fisher grade, gender, mode of aneurysm treatment, IPH, and type of SAH (aneurysmal versus peri-mesencephalic SAH) (Table 4). This study did not examine the role of dolichocarotids and the incidence of cerebral vasospasm.

## Discussion

In this study, we systematically examined short-term clinical and electrographic seizure incidences in SAH cohort in which a specific subgroup underwent early AED discontinuation. We found the incidence of pre-hospital seizures was 8% and the overall instance of sub-acute seizure post SAH was 1.1% with empiric AED use and 0% in eligible patients who underwent early AED discontinuation. We found that AED discontinuation was independently associated with good outcome at time of hospital discharge after adjusting for known predictors of functional outcome after SAH, and validated this logistic regression model using data partitioning. AED discontinuation was not associated with the incidence of angiographic vasospasm or with any increase in mortality. On the contrary, AED discontinuation appeared to be independently associated with lower in-hospital mortality on exploratory secondary analysis. We conclude from these analyses that early AED discontinuation is safe in SAH patients without prior seizure history who were awake and following commands after aneurysm intervention. The set of clinical criteria used in selecting this subset of patients was simple, standardized, and required no additional ancillary testing, therefore easily adaptable to many practice settings. In this cohort, early AED discontinuation in eligible SAH patients is independently associated with increased likelihood of discharge to home instead of another health care facility, which translates into potential reduction of healthcare-related cost by approximately 7% SAH patients [20]. In this study, we found 40% of all aneurysmal SAH admissions were eligible for AED discontinuation protocol. If we extrapolate to US wide, where 30,000 new cases of SAH occur yearly, this translates into 12,000 patients yearly who could potentially safely undergo early AED discontinuation therapy.

Observed incidences of SAH-related seizures in this study is lower than the 1.5% to 25% range reported in prior literature [5]. This is consistent with an observed decline in reported post-SAH seizure incidences over the past 20 years, possibly due to improvement in surgical techniques and new aneurysm treatment modalities [7,21,22]. Seizure incidences likely vary at different stages of SAH disease process, which may in part contribute to the wide range in reported SAH-associated seizure incidence. SAH disease course can be generally divided into three time periods: early (pre-hospital), subacute, and late. “Early” seizures typically refer to seizure-like clinical phenomena occurring at SAH onset prior to patient’s arrival to the hospital. It is unclear whether these events are true electrical seizures or neurological manifestation of sudden intracranial pressure (ICP) elevation secondary to aneurysm rupture. We observed 8% incidence of early seizures in our cohort, which is consistent with reported incidence of 7.8%-11% [23,24]. “Subacute” seizures typically refer to seizures occurring within 1-2 weeks after SAH onset and has a reported incidence of 1.5%-9.7% [6,25], higher in patients with thicker SAH clots [23,25,26], and with possibly up to 8% incidence of status epilepticus in comatose patients [27]. Sub-acute seizures in SAH have been the main target for prophylactic AED use, as seizures are historically thought to increase the risk of aneurysm re-rupture, which is associated with significant morbidity and mortality. AED-withdrawal protocol reported in this study specifically targets subacute seizures in SAH. We found that subacute seizure incidence in this patient cohort is lower than that reported in literature regardless of AED-withdrawal status. “Tate seizures” refer to seizures occurring months to years after SAH are and has a reported incidence of 4.9%-25% [21–22,28,29]. We did not examine late seizures in this study. Prior studies have demonstrated that prophylactic AED use did not appear to impact the incidence of late seizures following SAH [30].

Consistent with prior studies [7,10], our study also showed AED use is associated with worse outcome following SAH. Both prior studies used phenytoin, therefore it was not known whether prophylactic AED use in SAH is associated with poor outcome regardless of AED agent used, or whether the poor outcome is related to phenytoin effect. In fact, there is data to suggest that SAH patients treated with levetiracetam had better outcome compared to those treated with phenytoin [31]. On the other hand, there is also data to suggest that levetiracetam use in SAH may be associated with higher rate seizures compared to phenytoin [32]. In this study, over 50% of our SAH cohort was initially exposed to phenytoin and then switched to levetiracetam monotherapy after aneurysm treatment. Despite prevalent use of levetiracetam as AED of choice, our study still showed a strong independent association between prophylactic AED use and poor SAH outcome. It is possible that phenytoin exposure, even for a very short time, can lead to poor outcome after SAH. Alternatively, our data can also be consistent with the hypothesis that prophylactic use of either phenytoin or levetiracetam in all SAH patients regardless of seizure risk can lead to poor outcome. A prospective randomized study is necessary to answer this question.

Results from this study must be interpreted with consideration of several limitations. This is a cohort study where two potentially dissimilar groups were compared. Though known important predictors of SAH outcome were comparable between groups and though we have adjusted for important confounders and validated our result using data partition, there may still be unknown confounders not accounted for in this study design. It is possible that clinical features associated with meeting the inclusion and exclusion criteria for early AED discontinuation are themselves associated with better outcome after SAH despite adjusting for HH score, age, vasospasm, infarct, IPH, and IVH. Though we are able to demonstrate a robust and strong association between early AED discontinuation and good functional outcome, this study does not address the issue of causality. A prospective randomized study is necessary to establish the causal relationship between empiric AED discontinuation and clinical outcome. Secondly, our study did not prospectively record data on potential sub-clinical seizures in this patient population. However, if patients who underwent early AED discontinuation had increased incidence of sub-clinical seizures, these seizures likely did not affect their outcome given that we found early AED discontinuation is associated with lower mortality and better short term functional outcome. Finally, there is no long-term followup data from this cohort study. The scope of this study does not address the long-term effect of early AED discontinuation on SAH outcome and epileptogenesis.

Data from this study support clinical protocols for early AED discontinuation in SAH patients who are awake and following commands. The association between early AED discontinuation and better short-term functional outcome in this study provides further support for possible deleterious effects of empiric AED use in SAH. Together, these data suggest a clear need for a prospective randomized clinical trial to determine the effect of empiric AED use in SAH. Results from this study also lead to the important question of whether empiric AED discontinuation may be safe, feasibly and beneficial in SAH patients who did not meet inclusion criteria for AED discontinuation in this study. The low overall incidence of sub-acute seizures post SAH and the correlation between AED use and poorer functional outcome in several studies support clinical equipoise in this question. A prospective study of this subset of patients with continuous EEG monitoring would be necessary to establish the safety and feasibility of AED discontinuation in a SAH cohort with poor mental status.

## Figures and Tables

**Table 1:** Baseline Patient Characteristics

	OFFAED (n=73)	ON AED (n=93)	P
**Gender (# female)**	46 (63%)	66 (71%)	0.28*
**Mean Age (years)**	55	54.4	0.77^†^
**Surgical clipping**	44 (60%)	55 (59%)	0.9*
**Hunt & Hess** HH1 HH2 HH3 HH4 HH5	Median=2	Median=2	0.01*
	33 (45%)	38 (41%)	
	25 (34%)	11 (12%)	
	7 (9.6%)	19 (20%)	
	4 (5.5%)	12 (13%)	
	4 (5.5%)	13 (14%)	
**Fisher Grade** F0 F1 F2 F3 F4	Median=3	Median=3	
	0 3 (4.1%) 12 (16%) 52 (71%) 6 (8.2%)	0 5 (5.4%) 7 (7.5%) 77 (83%) 4 (4.3%)	0.19*
**Intraventricular Hemorrhage**	39 (53%)	55 (59%)	0.46*
**Intraparenchymal Hemorrhage**	12 (16%)	20 (22%)	0.41*
**Angiographic Vasospasm**	21 (30%)	36 (39%)	0.25*
**Ischemic Infarct**	14 (20%)	13 (14%)	0.37*
**External Ventricular Drain Insertion**	31 (42%)	52 (56%)	0.09*
**Aneurysm Location**			0.71*
**Anterior Circulation**	50 (68%)	65 (70%)	
Anterior communicating	16 (22%)	26 (29%)	
Middle cerebral	8 (11%) 6 (8.2%)	13 (14%) 3 (3.2%)	
Anterior cerebral			
Internal carotid	6 (8.2%)	8 (8.6%)	
Posterior communicating	13 (18%)	13 (14%)	
Opthalmic	1 (1.4%)	1 (1.1%)	
Anterior choroidal	0	1 (1.1%)	
**Posterior Circulation**	12 (16%)	21 (23%)	
Basilar	7 (9.6%)	12 (13%)	
Vertebral	0	2 (2.2%)	
Superior cerebellar	2 (2.7%)	2 (2.2%)	
Posterior inferior cerebellar	3 (4.1%)	5 (5.4%)	
**Angiogram-negative SAH**	10 (14%)	7 (7.5%)	
**Multiple aneurysms**	1 (1.4%)	0	

(*: Chi-square Test, ^†^: 2-tailed T-test).

**Table 2:** Anticonvulsant Agent Exposure in SAH Patients Receiving Empiric Anticonvulsants.

Anticonvulsant Agent	Number of subjects
Phenytoin	33 (35%)
Levetiracetam	2 (2.1%)
Phenytoin switched to levetiracetam	54 (58%)
Phenytoin and levetiracetam	2 (2.1%)
Phenytoin switched to phenobarbital	1 (1.1%)
Phenytoin and gabapentin	1 (1.1%)

**Table 3:** Clinical Outcomes after Early Anticonvulsant (AED) Discontinuation versus Empiric AED Use

Outcome	OFF AED (n=73)	ON AED (n=93)
**Seizure**	0	1 (1.1%)
Mean ICU LOS* (days)	10.3	10.3
**Mean Hospital LOS (days)**	16.6	15.7
**Mortality**	2 (2.7%)	22 (24%)
**Discharge to home**	43 (59%)	27 (29%)

(* LOS: length of stay).

**Table 4:** Logistic Regression Models of Discharge Outcome, Mortality, and Angiographic Vasospasm.

Parameter	Estimate	standard error	pr > chisq
**Model: Discharge Outcome**			
Intercept	4.81	1.81	0.01
*AED discontinuation*	1.02	0.32	0.002
Hunt and Hess Score	−0.75	0.28	0.01
Age	−0.09	0.03	0.0013
Vasospasm	−1.03	0.35	0.004
Infarct	−1.03	0.45	0.02
IPH	−0.64	0.43	0.14
IVH	−0.60	0.31	0.06
**Model: Mortality**			
Intercept	−4.7	1.54	0.0021
*AED discontinuation*	−3.15	1.05	0.0028
Hunt and Hess Score	1.38	0.3	<0.0001
age	−0.0029	0.02	0.9
Vasospasm	−2.25	0.86	0.009
Infarct	0.23	0.86	0.79
IPH	−1.56	0.88	0.08
IVH	1.17	0.87	0.18
**Model: Vasospasm**			
Intercept	−1.74	1.39	0.21
*AED discontinuation*	−0.12	0.39	0.76
age	−0.05	0.02	0.0023
Hunt and Hess Score	0.55	0.17	0.001
Fisher Grade	0.48	0.39	0.22
Perimesencephalic SAH	2.32	1.34	0.08
gender	0.29	0.41	0.48
Aneurysm treatment -coil (versus craniotomy)	0.49	0.47	0.30
Aneurysm treatment – none (versus craniotomy)	−2.25	0.78	0.004
IPH	−0.51	0.49	0.30
